# Perspectives of Bacterial PAPS-Independent Aryl Sulfotransferases for Practical *In Vitro* Sulfation

**DOI:** 10.1021/acs.jafc.6c01204

**Published:** 2026-07-16

**Authors:** Katerina Brodsky, Barbora Petránková, Kristýna Slámová, Pavla Bojarová, Kateřina Valentová

**Affiliations:** 86863 Institute of Microbiology of the Czech Academy of Sciences, Vídeňská 1083, 142 00 Prague, Czech Republic

**Keywords:** aryl sulfotransferases (ASTs), Desulfitobacterium hafniense, *in vitro*, sulfation, (poly)phenols

## Abstract

PAPS-independent bacterial aryl sulfotransferases (ASTs) do not require the costly and unstable cofactor PAPS like mammalian sulfotransferases. Instead, they use simple aromatic sulfuryl donors. Originally discovered in intestinal bacteria, ASTs display remarkable substrate diversity, catalyzing sulfation of phenols, alcohols, amines, sugars, and polyphenols, including flavonoids and flavonolignans. Among them, AST from *Desulfitobacterium hafniense* (*Dh*AST) is particularly notable for its stability and broad substrate range. Structural and mechanistic studies reveal that ASTs follow a ping-pong bibi mechanism with transient enzyme sulfation. Recent identification of new ASTs from diverse bacterial species and advances in recombinant expression have broadened the potential of these enzymes for selective and scalable synthesis of sulfated metabolites *in vitro*. Expanding the available AST library has deepened the understanding of bacterial sulfation pathways and supports their applications in biocatalysis, metabolite synthesis, and production of sulfated bioanalytical standards.

## Graphical abstract

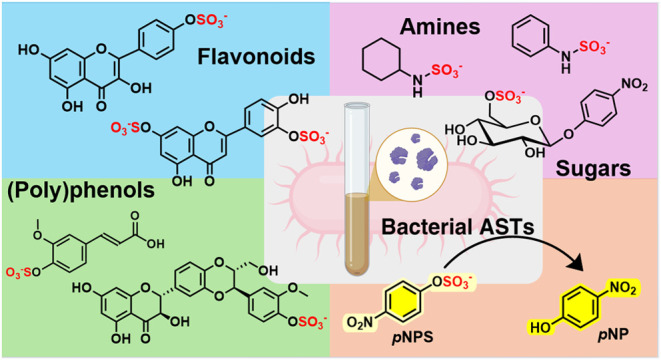

## Introduction

The biological activity of many molecules *in vivo* is closely linked to their sulfation pattern. During the biotransformation of xenobiotics in humans, sulfation represents a major phase II metabolic pathway that regulates the activity, solubility, and excretion of a wide range of compounds.^1,2^ A substantial proportion of these xenobiotics are natural (poly)­phenols, widely present in vegetables, fruits, grains, and seeds. These phytochemicals are an integral part of the human diet, and their sulfation plays a crucial role in their metabolic fate.^3^ However, (poly)­phenols are rapidly transformed *in vivo*, and their circulating forms predominantly comprise conjugated metabolites, including sulfates, which are often difficult to detect, isolate, and study in biological systems.^4^

Sulfation can significantly alter the physicochemical and biological properties of (poly)­phenols, including their stability, bioavailability, and interactions with molecular targets.^5–7^ Importantly, sulfated metabolites may exhibit distinct biological activities that are sometimes not directly predictable from their parent compounds.^8–14^ These differences complicate the interpretation of the health effects of dietary (poly)­phenols, as the compounds present in circulation and tissues are largely their conjugated forms rather than the native molecules.^15^ Consequently, access to structurally well-defined sulfated metabolites is essential for reliable bioactivity evaluation and metabolic profiling.^16,17^ However, the isolation of these metabolites from the biological material is challenging, and their stability is often limited.

*In vitro* sulfation of polyphenolic compounds represents a controlled and efficient route to obtain these metabolites, to be used as analytical standards and tools for biological investigations. Chemical sulfation, however, often suffers from poor selectivity, low stability of the products, and contamination with inorganic salts, which are difficult to remove and may interfere with downstream applications.^18^ Silica gel chromatography, commonly used for purification, is generally unsuitable due to the high polarity of sulfated compounds. Furthermore, regioselective sulfation of hydroxyl or amino groups is difficult to achieve chemically,^19^ in contrast to enzymatic syntheses that are more regioselective and proceed under mild conditions.

Enzymatic sulfation can be efficiently accomplished using sulfotransferases (EC 2.8.2) that catalyze the transfer of a sulfuryl group (SO_3_^–^) from a donor molecule to an acceptor alcohol or amine, producing water-soluble sulfuric acid esters: sulfates (OSO_3_^–^) when transferred to an alcohol, or sulfamates (NHSO_3_^–^) when transferred to an amine.^20^ In mammals, sulfation of both endogenous compounds and xenobiotics is catalyzed by two basic enzyme types. Golgi-associated, membrane-bound sulfotransferases primarily modify endogenous peptides, proteins, and glycosaminoglycans. A significant member of this group is *N*-deacetylase/*N*-sulfotransferase, an essential enzyme in heparan sulfate (HS) biosynthesis.^21,22^ In contrast, cytosolic sulfotransferases (SULTs),^23^ freely present in the cytosol, primarily catalyze the sulfation of small endogenous molecules and xenobiotics.^24,25^ SULTs require the cofactor 3′-phosphoadenosine-5′-phosphosulfate (PAPS) as a universal sulfuryl donor, which classifies them as PAPS-dependent sulfotransferases. They have two main binding sites: the active site and the PAPS binding site. The PAPS binding site is conserved in all SULTs, while the active site exhibits considerable variability.^2^ SULTs are highly regioselective and play a crucial role in the sulfation of compounds such as estradiol, thyroid hormones, steroids, and drugs in the human body.^1,20,26,27^ However, the required cofactor, PAPS, is unstable and very expensive, and some recombinant SULTs are also unstable in the *in vitro* environment.^28,29^ Therefore, this type of enzyme is impractical for the routine preparation of sulfated products with high yields on a preparative scale.^30^

PAPS-independent aryl sulfotransferases (ASTs, EC 2.8.2.22), which utilize simple aromatic sulfuryl donors, are more promising for potential laboratory use.^31–35^ When first described, these enzymes were named “aryl sulfate sulfotransferases” (ASSTs). Over the past two decades, the naming of these enzymes has varied across publications. In this review, the abbreviation ASTs refers to the PAPS-independent aryl sulfotransferases. These mostly bacterial enzymes transfer sulfuryl groups from donors to acceptors without PAPS mediation (Figure 1), offering new perspectives for the sustainable synthesis of sulfated compounds. Moreover, they are easily heterologously expressed in *Escherichia coli* and tend to be more stable than SULTs.^30^

**Figure 1:**
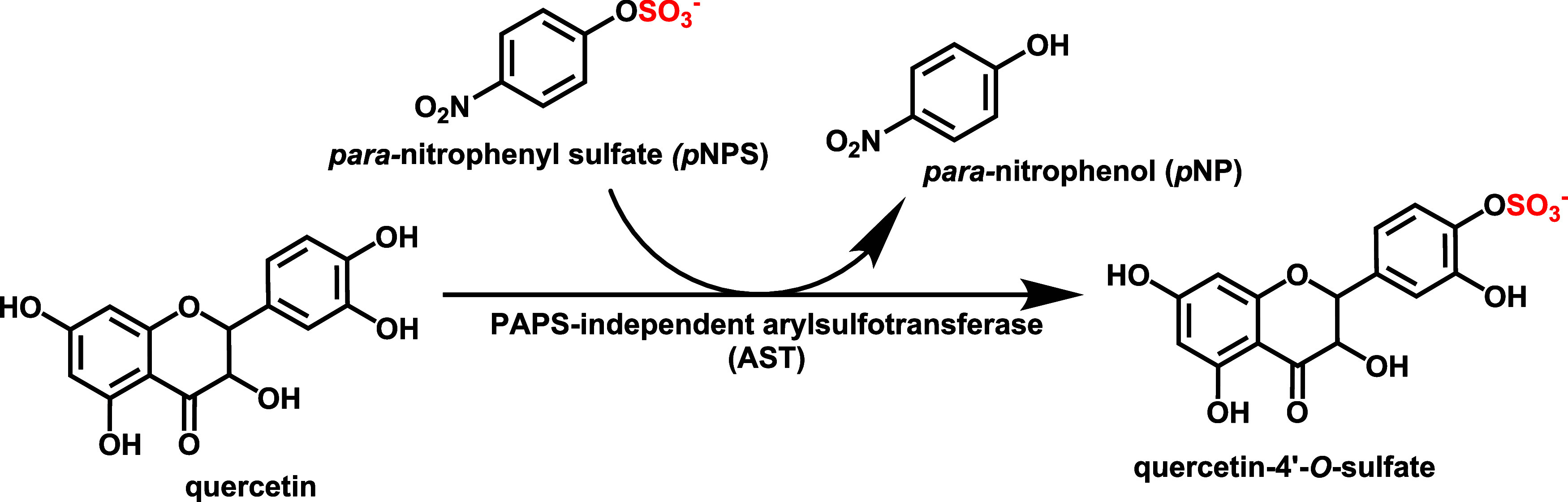
Sulfation of quercetin by a PAPS-independent aryl sulfotransferase (AST).

Even though the first AST was described in 1975, only a handful of ASTs have been characterized since then, and the most recent review was published 15 years ago, focusing mainly on the enzyme structure and mechanism.^36^ In the intervening years, new ASTs have been discovered, and new acceptors have been investigated—not only phenols, but also alcohols, amines, polyphenols and their derivatives, sugars, and others. Additionally, new sulfuryl donors have been proposed and tested, offering alternatives to those previously known. The aim of this review is to present an updated overview of the properties and substrate preferences of currently known bacterial ASTs. We hope it will serve as a useful guide to ASTs and their practical applications in the production of sulfated compounds.

## Microbial PAPS-Independent ASTs

The first putative PAPS-independent aryl sulfotransferase (AST, Table 1) was identified in the crude extract of *Aspergillus oryzae* during a study on its sulfatases (I–III).^37^ In the fractions containing aryl sulfatase II, a significant increase in the hydrolysis of *p*-nitrocatechol sulfate was observed upon the addition of tyramine. It was suggested that “aryl sulfatase” II acts as a transferase *in vivo*, despite exhibiting characteristics of a typical aryl sulfatase in the absence of an external sulfuryl acceptor. This enzyme was unable to utilize PAPS as a sulfuryl donor. Instead, it showed substrate preference for *p-*acetylphenyl sulfate, followed by 4-methylumbelliferyl sulfate (MUS) and *p*-nitrophenyl sulfate (*p*NPS).^37^ The kinetic data and the presence of free sulfate indicated the formation of an intermediate enzyme-sulfate complex, suggesting a ping-pong reaction mechanism. In the aryl sulfatase activity of this enzyme, water probably replaces the phenol acceptor and degrades the enzyme-sulfate intermediate by the same mechanism.^38^

**Table 1:** Published PAPS-Independent Aryl Sulfotransferases, Their Biochemical Properties, and Substrate Specificity for Sulfuryl Donors and Acceptors

source of AST	pH	temperature [°C]	donors	acceptors	refs
*Aspergillus oryzae*	8–9	45–55	*p*-nitrocatechol sulfate	*N*,*N*-dimethyltyramine, phenol, *p*-hydroxyacetophenone, *p*-hydroxyphenylpropionic acid, *p*NP, tyramine, tyrosine, vanillin	^37,38^
			*p*-acetylphenyl sulfate		
			*p*NPS		
*Eubacterium A-44*	8–9	<45	estrone sulfate, indoxyl sulfate, MUS, *p*-acetylphenyl sulfate, picosulfate, *p*NPS	2,4,6-trihydroxybenzoic acid, 3,5-dihydroxybenzoic acid, 4-methylumbelliferone, angiotensin III, Arg-vasopressin, butylparaben, CCK-8-NS, DOPA, dopamine, epinephrine, estradiol, insulin, kyotorphin, Leu-enkephalin, *p*-cresol, LH-RH, Met-enkephalin, methylparaben, ethylparaben, propylparaben, butylparaben, *o*-; *m*-; *p*-acetamidophenol, *p*-hydroxybenzoate ethylester, *p*-hydroxyphenylpropionic acid, phenol, proctorin, propylparaben, salicylamide, tyramine, tyrosine methylester, α-; β-naphthol	^40–42,68,69^
*Klebsiella K-36*	10.0–10.5	<50	estrone sulfate, MUS, *p*-acetylphenyl sulfate, *p*NPS	2,4,6-trihydroxybenzoic acid, 3,5-dihydroxybenzoic acid, 9-phenanthrol, butylparaben, CCK-8-NS, Kyotorphin, Leu-enkephalin, *o*-; *m*-; *p*-chlorophenol, methylparaben, ethylparaben, propylparaben, butylparaben, *p*-aminophenol, phenol, *p*-hydroxyphenylpropionic acid, tyramine, tyrosine, α-naphthol	^46,68^
*Haemophilus K-12*	8–9	NA^a^	MUS, *p*NPS	2,4,6-trihydroxybenzoic acid, 2,4-dihydroxybenzoic acid, 3,4-dihydroxybenzoic acid, 3,5-dihydroxybenzoic acid, estrone, kyotorphin, Leu-enkephalin, *m*-aminophenol, naringin, methylparaben, ethylparaben, propylparaben, butylparaben, phenol, *p*-acetoaminophen, α-naphthol, *p*-hydroxyphenylpropionic acid, phloroglucinol, resorcinol, serotonin, tyramine, tyrosine	^45,68^
			α-; β-naphthyl sulfate		
*Enterobacter amnigenus* *AR-37*	9	NA^a^	MUS, *p*NPS, α-; β-naphthyl sulfate	*p*-acetaminophen, phenol, resorcinol, tyramine, tyrosine, α-naphthol	^50^
*Eubacterium reclate* IIIH	NA^a^	NA^a^	MUS, *p*NPS	tyramine	^51^
*Citrobacter freundii*	NA^a^	NA^a^	*p*NPS	*p*-acetaminophen, phenol, resorcinol, tyramine, tyrosine, α-naphthol	^52^
*Salmonella typhimurium*	NA^a^	NA^a^	*p*NPS	*p*-acetaminophen, phenol, resorcinol, tyramine, tyrosine, α-naphthol	^32^
*Clostridium innocuum*	8.2	NA^a^	*p*-nitrocatechol sulfate, estrone sulfate, indoxyl sulfate, MUS, *p*-acetylphenyl sulfate, *p*NPS, phenolphtalein sulfate	2,2’-bisphenol, 2,4-dichlorophenol, 2-ethylphenol, 2-isopropylphenol, 2-propylphenol, 2-(*sec*-butyl)phenol, 3,5-dimethylphenol, 3-ethylphenol, 4,4’-bisphenol, 4-acetamidophenol, 4-aminophenol, 4-chlorophenol, 4-methylumbelliferone (MU), catechin 8-hydroxyquinoline, benzoquinone, catechol, o-; *m*-; *p*-cresol, phenol, tyramine, tyrosine containing peptides (physalaemin, kyotorphin, dermorphin, proctolin, Leu enkephalin, cholecystokinin, angiotensin I, caerulein, Arg-vasopressin), tyrosine methyl ester	^70^
*Escherichia coli* *CFT073 (Ec*AST*)*	8	NA^a^	MUS, *p*NPS	apigenin, caffeic acid, catechol, chrysin, ferulic acid, fisetin, genistein, hesperetin, kaempferol, luteolin, myricetin, phenol, quercetin	^33,61^
*Streptomyces sp. MK730–62F2*			MUS, *p*NPS	caprazamycin (Cpz4), phenol	^71^
*Desulfitobacterium hafniense* *(Dh*AST*)*	9	40	4-acetylphenyl sulfate, MUS, *N*-hydroxysuccinimide-sulfate, *N*-phtalimide sulfate, *o*-; *m*-; *p*-NPS, phenyl sulfate	1,3,5-benzenetriol, 17α-bihydroequiline, 17α-estradiol, 17β-estradiol, 1-butanol, 1-hexanol, 1-ctanol, 1-pentanol, 2,2‘-bisphenol, 2,3-dehydrosilybin, 2,3-dehydrosilychristin, 2-hydroxyphenylacetic acid, 2-methoxyestradiol, 2-phenylethyl alcohol, 2-phenylphenol, caffeic acid 3-(4-hydroxyphenyl)propionic acid, 3,4-dihydroxyphenylacetic acid, 3,5-dimethoxyphenol, 3-bromocatechol, 3-fluorocatechol, 3-hydroxyphenylacetic acid, 3-methoxycatechol, 3-methylcatechol, 4,4‘-dihydroxybiphenyl, 4-hydroxyindole, 4-hydroxyphenylacetic acid, 5-hydroxyindole, 6-hydroxyflavone, ampelopsin, aniline, 2-aminocyclohexanol, 2-methoxycyclohexanol, cyclohexanediol, apigenin, benzenetriol, benzyl alcohol, bisphenol A, catechol, cyclohexanol, dopamine, estrone, ferulic acid, fisetin, furfuryl alcohol, gallic acid, genistein, glycerol, hesperetin, hydroquinone, chrysin, hydroxytyrosol, kaempferol, Leu-enkephalin, luteolin, *m*-chlorocatechol, *m*-; *p*-chlorophenol, myricetin, *N*-acetylglucosamine, phenol, *N*-hydroxysuccinimide, o-; *p*-aminophenol, *p*-cresol, *p*-coumaric acid, phloretin, rutin, tyrosol, taxifolin, tyramine, *p*NP-glycerol, *p*NP-β-d-glucopyranoside, quercetin, phenyl-β-d-glucopyranoside, 1,5-hexanediol, 2-hexanol, resorcinol, resveratrol, isoquercitrin, silychristin, silybin A, silybin B, isosilybin A, isosilybin B, uridine, α-; β-naphthol	^8,30,34,35,55–61,72–75^
*Desulfosporosinus sp. HMP52 (Ds*AST*)*	9.5	40–55	*p*NPS	apigenin, caffeic acid, catechol, chrysin, ferulic acid, fisetin, genistein, hesperetin, kaempferol, luteolin, myricetin, phenol, quercetin	^61^
*Desulfofalx alkaliphila* *(Dal*AST*)*	8.5	55	*p*NPS	apigenin, caffeic acid, catechol, chrysin, ferulic acid, fisetin, genistein, hesperetin, kaempferol, luteolin, myricetin, phenol, quercetin	^61^
*Salmonella bongori* *(Sb*AST*)*	10	50	*p*NPS	apigenin, caffeic acid, catechol, chrysin, ferulic acid, fisetin, genistein, hesperetin, kaempferol, luteolin, myricetin, phenol, quercetin	^61^
*Campylobacter fetus* *(Cf*AST*)*	12	55	*p*NPS	apigenin, caffeic acid, catechol, chrysin, ferulic acid, fisetin, genistein, hesperetin, kaempferol, luteolin, myricetin, phenol, quercetin	^61^
*Desulfitobacterium dehalogenans*	5	45	*p*NPS	1,3,5-benzenetriol, 1-hexanol, 4,4’-dihydroxybiphenyl, 2-aminocyclohexanol, 2-methoxycyclohexanol, cyclohexanediol, aniline, benzenetriol, catechol, cyclohexanol, cyclohexylamine, hydroquinone, 2-hexanol, *N*-acetylglucosamine, phenol, 1,5-hexanediol, resorcinol, uridine, β-naphthol	^62^
*Dehalobacterium formicoaceticum*	8	40	*p*NPS	1,3,5-benzenetriol, 1-hexanol, 4,4‘-dihydroxybiphenyl, 2-aminocyclohexanol, 2-methoxycyclohexanol, cyclohexanediol, aniline, benzenetriol, catechol, cyclohexanol, cyclohexylamine, hydroquinone, *N*-acetylglucosamine, phenol, 1,5-hexanediol, 2-hexanol, resorcinol, uridine, β-naphthol	^62^
*Hungatella effluvii*	8	25	*p*NPS	1,3,5-benzenetriol, 1-hexanol, 2-hexanol, cyclohexanol,4,4‘-dihydroxybiphenyl, 2-aminocyclohexanol, 2-methoxycyclohexanol, cyclohexanediol, aniline, benzenetriol, catechol, hydroquinone, *N*-acetylglucosamine, phenol, 1,5-hexanediol, resorcinol, uridine, β-naphthol	^62^
*Desulfosporosinos orientis*	9	45	*p*NPS	1-hexanol, phenol, 4,4‘-dihydroxybisphenyl, 2-aminocyclohexanol, 2-methoxycyclohexanol, cyclohexanediol, aniline, benzenetriol, catechol, cyclohexanol, hydroquinone, *N*-acetylglucosamine, 1,5-hexanediol, 2-hexanol, resorcinol, β-naphthol	^62^
*Bacteroides vulgatus*	NA^a^	NA^a^	acetaminophen sulfate, dopamine sulfate, indoxyl sulfate, MUS, *p*-cresol sulfate, *p*-coumaric acid sulfate, *p*NPS	4-ethylphenol, dopamine, acetaminophen, 4-hydroxyphenylpyruvic acid, caffeic acid, *o*-; *m*-; *p*-cresol, *p*-coumaric acid, phenol, *p*NP, tyramine, β-naphtol	^66^

a Information is not available. *p*NPS = *p*-nitrophenyl sulfate. MUS = methylumbelliferyl sulfate.

The first reported bacterial AST was found in *Eubacterium* A-44 (Table 1), isolated from human intestinal microbiota. It was hypothesized that the sulfuryl groups of phenolic sulfate esters in the gut are rapidly transferred to other phenols by the gut microbiota, indicating the vital role of sulfation in the metabolism and detoxification of phenolic compounds.^39^ In contrast to the previously described enzyme from *A. oryzae*, this AST demonstrated strict sulfotransferase activity without hydrolyzing the sulfuryl donor. The production of AST in *Eubacterium* A-44 was promoted by the addition of donor substrates, such as *p*NPS, to the culture medium. Similar to *A. oryzae*, AST from *Eubacterium* A-44 exhibited unique acceptor specificity that differed from SULTs and did not utilize PAPS as a donor.^40^ Over the following decade, AST from *Eubacterium* A-44 was extensively studied in several publications.^31,40–43^ Later, the enzyme was produced heterologously in *E. coli* BL21­(DE3) as a His-tagged construct, resulting in a higher activity and production levels than the wild-type enzyme.^44^

Many studied ASTs were isolated from mammalian intestinal microbiota, mostly from the human gut. Aryl sulfotransferase from *Haemophilus* K-12 ^45^ (Table 1) was identified in the gut microbiota of a mouse, and AST from *Klebsiella* K-36 was observed and isolated from the intestinal microbiota of rats.^46^ The gene encoding for AST from *Klebsiella* K-36 (*ast*A, GenBank accession U32616.1) was heterologously expressed in *E. coli* NM522 in 1996, making it the first recombinant AST.^47^ The cell lysate of the recombinant AST showed approximately a 12-fold increase in sulfotransferase activity compared to the cell lysate of *Klebsiella* K-36. The purified recombinant AST exhibited kinetic properties similar to those of the wild-type enzyme. The *ast*A gene from *Klebsiella* K-36 contains a sequence of a signal peptide that directs the enzyme into the periplasm. To increase protein yield and simplify purification, this signal peptide was removed, and *ast*A was fused with the gene of glutathione *S*-transferase (GST) for coexpression. After expression and cleavage of the GST-AST fusion protein, AST retained its high specific activity. These results confirmed that *ast*A can act as a reporter gene since the activity of the expressed AST was independent of a foreign gene.^48,49^

Several other recombinant ASTs derived from the gut microbiota were produced and characterized, including those from *Enterobacter amnigenus* AR-37,^50^ *Eubacterium rectale* IIIH,^51^ *Citrobacter freundii*,^52^ and *Salmonella typhimurium* (Table 1).^32^ Sequence analysis of each of these ASTs, and the one from *Klebsiella* K-36, revealed two contiguous open reading frames (ORF1 and ORF2) on the same strand. Based on amino acid sequence homology, the genes were identified as *astA* (encoding for AST) and *dsbA* (encoding for a thiol–disulfide oxidoreductase, DsbA). DsbA is an enzyme involved in forming disulfide bonds in proteins within the periplasmic space,^53^ and provides a signal peptide that directs the AST to the periplasm.^12,28–32^

A similar phenomenon was observed in the AST from uropathogenic *E. coli* CFT073 (Table 1). This *E. coli* strain possesses an additional homologous protein system, DsbL/DsbI, encoded in the same operon as a periplasmic AST, resulting in the coexpression of all three proteins. The AST from *E. coli* CFT073 (*Ec*AST), one of the largest proteins in the periplasm, contains a single disulfide bond essential for its activity and serves as a substrate for the DsbL/DsbI system *in vivo*. Therefore, efficient production of functional AST requires the DsbL/DsbI system.^54^ Among all published ASTs, *Ec*AST appears to be the only one with a confirmed disulfide bond in its structure.

One of the most studied bacterial aryl sulfotransferases is AST from *Desulfitobacterium hafniense* (*Dh*AST, Table 1).^34^ Notably, it is the first AST shown to sulfate not only small aromatic alcohols but also a variety of aliphatic alcohols, sugars, steroids, and polyphenols.^8,34,55–60^ In addition to the recombinant wild-type enzyme, a *Dh*AST variant with a polyhistidine tag was produced. This modification facilitated enzyme purification and enhanced its activity.^15^ Advantageously, due to its high stability and absence of sulfatase activity in the crude cell lysate, *Dh*AST can be used directly without purification.^60^

The substrate variability of *Dh*AST has evoked interest in identifying new potential ASTs. Sequence homology analysis based on the amino acid sequences of known ASTs revealed a large group of new putative ASTs.^61,62^ Several of them were expressed and studied: ASTs from *Desulfosporosinus* sp. HMP52 (*Ds*AST), *Desulfofalx alkaliphile* (*Dal*AST), *Salmonella bongori* (*Sb*AST), *Campylobacter fetus* (*Cf*AST), *Desulfitobacterium dehalogenans*, *Dehalobacterium formicoaceticum*, *Hungatella effluvia*, *Desulfosporosinus orientis*, and a yet unknown variant of AST from *D. hafniense* (Table 1).

Computational approaches are gaining increased attention and application in protein research. Bioinformatic analyses have identified putative ASTs in the microbial genus *Sutterella*, found in the human gut, which have been associated with various health conditions.^63,64^ Seventeen different genes potentially encoding for ASTs were identified and characterized in *Sutterella wadsworthensis* (*Swa*AST) by sequence analysis and structural prediction. Although sequence analysis^35^ grouped *Ec*AST and *Swa*AST into the same cluster based on their shared sequence features, *Swa*AST shares only approximately 53% sequence identity with *Ec*AST. Such a substantial divergence suggests that both enzymes may possess distinct structural and functional properties.^65^

The most recent study describes a novel AST from the prevalent gut bacterium *Bacteroides vulgatus* (referred to as *Bv*AST here, Table 1). This enzyme was identified through gene mining of sequenced gut microbiota genomes and fecal samples from patients. Notably, *B. vulgatus* possesses two sulfotransferase-encoding genes: one predicted to encode for a PAPS-dependent sulfotransferase and the other identified as a putative AST. Recombinant *Bv*AST demonstrated a broad substrate specificity for the tested phenolic acceptors and donors.^66^

## Structure and Function of ASTs

SULTs have been shown to follow a sequential bi–bi catalytic mechanism, requiring both substrates to interact with the enzyme for the reaction to proceed. Studies of known human (hSULTs) and other mammalian SULTs indicate that this bi–bi mechanism is not consistent across all sulfotransferases (Figure 2A).^24^ Most are described as ordered reactions, while some follow a random mechanism. In ordered bi–bi reactions, the sequence in which substrates bind to the active site is critical. The first substrate typically induces a conformational change in the enzyme, allowing the second substrate to bind. This mechanism aligns with the role of the cofactor PAPS. The cofactor binding site is conserved in most SULTs (*e.g.*, in hSULT1E1: Lys47, Lys105, His107, and Ser137). In this context, His acts as the catalytic base. The two Lys residues stabilize the transition state during SO_3_^–^ group transfer, while Ser is essential for stabilizing the 3′-phosphate of PAPS and for forming a hydrogen bond with Lys47 in the absence of an acceptor substrate, thus preventing hydrolysis of PAPS.^20^

**Figure 2:**
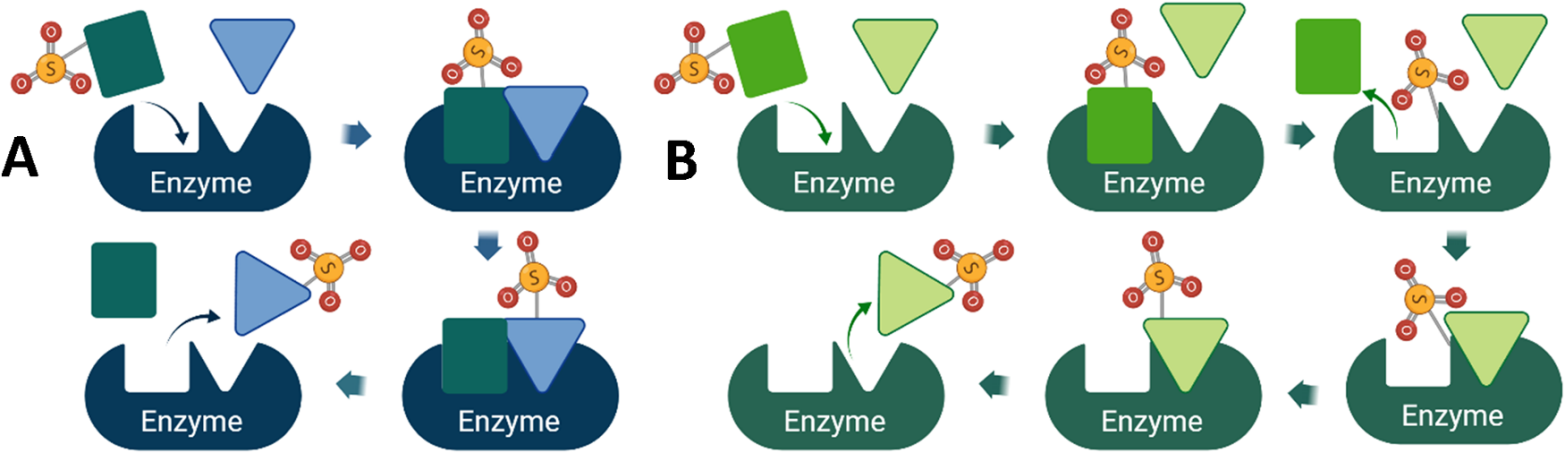
Catalytic mechanisms of enzymatic reactions. (A) Catalytic mechanism of the bi-bi reaction in PAPS-dependent sulfotransferases. Both substrates must bind to the active site simultaneously and the sulfuryl transfers from the donor to the acceptor. (B) Ping-pong bi-bi catalytic mechanism of bacterial aryl sulfotransferases. The donor molecule transfers the sulfuryl to the enzyme; the enzyme subsequently transfers the sulfuryl to the acceptor molecule.

Bacterial ASTs follow a different mechanism; they have been shown to react *via* a ping-pong bi–bi catalytic mechanism (Figure 2B), as demonstrated by Kim et al.^40^ in a study on AST from *Eubacterium* A-44. Using a labeled sulfuryl donor (^35^S)-*para*-nitrophenyl sulfate, an enzyme-donor intermediate complex was isolated and analyzed. The sulfate-containing amino acid in the intermediate was identified as tyrosine. According to the proposed mechanism, the reaction begins with the binding of *p*NPS to a histidine residue, forming an unstable intermediate and releasing *p*NP.^31,42,43^ The sulfuryl group is then transferred to a neighboring tyrosine residue, forming a stable intermediate. The tyrosine residue subsequently rapidly transfers the sulfuryl moiety to the acceptor, such as tyramine. In the study, the enzyme was irreversibly inactivated by several histidine-modifying agents, supporting the hypothesis that a histidine residue serves as an anion recognition site in the enzyme’s active site (Figure 3A).^42^ Earlier sequence analyses of several ASTs revealed conserved regions, particularly 11 conserved tyrosine residues likely to be part of the AST active site.^32^ Using site-directed mutagenesis, each conserved tyrosine residue in AST from *Enterobacter amnigenus* was individually replaced with phenylalanine to minimize conformational changes. Based on results with this enzyme containing the conserved regions, tyrosine at position 123 was identified as the key amino acid in the active site of AST.^67^

**Figure 3:**
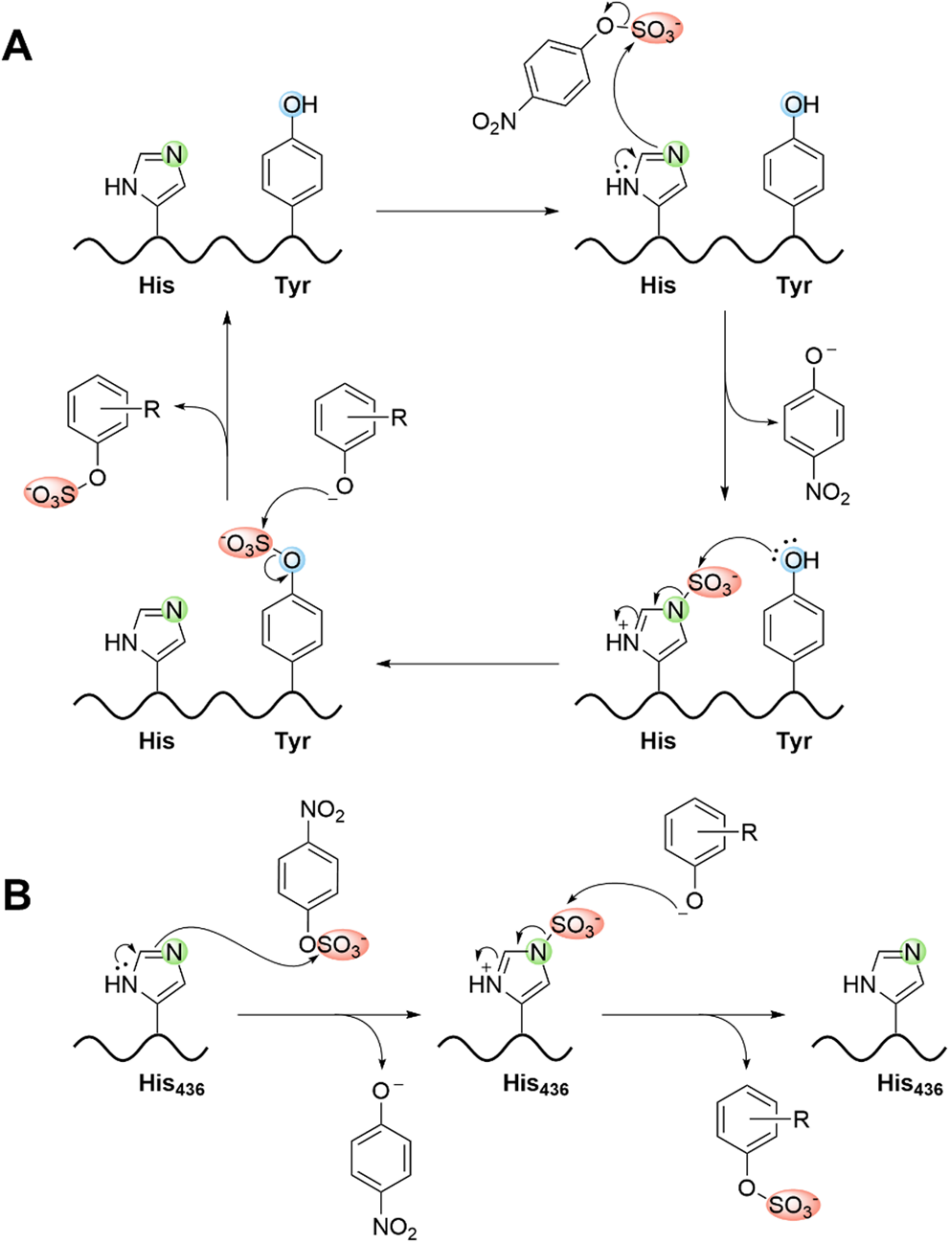
Reaction mechanisms proposed for ASTs. (A) The proposed reaction mechanism and active site of ASTs from *Eubacterium* A-44. (B) The active site and enzymatic reaction of *Ec*AST, as determined from the crystal structure.

Today, *Ec*AST is the only AST with a resolved crystal structure. X-ray crystallography (PDB: 3ELQ) has revealed that it is a homodimeric protein, with each monomer (approximately 63.8 kDa) consisting of an N-terminal 7-stranded β-sandwich domain and a larger C-terminal 6-bladed β-propeller domain.^54^ Additional analysis of *Ec*AST with the donors *p*NPS and MUS (PDB: 3ETS and 3ETT) indicated that the enzyme binds substrates in a 1:1 stoichiometry and catalyzes sulfuryl transfer *via* a ping-pong reaction mechanism involving transient sulfation of His436, a previously unknown covalent modification in proteins. In line with this, sulfation on histidine was recently also shown for *Bv*AST.^66^ In contrast, previous studies suggest that AST catalysis involves covalent modification of a tyrosine residue in the active site, and some propose a catalytic pair of tyrosine and histidine.^43,46,68^ However, the crystal structure of *Ec*AST shows that Tyr96 is distant from the active site and is not involved in catalysis in this AST. *Ec*AST catalysis appears to be a two-step process.^33^ In the first step, AST is covalently sulfated at His436, and the desulfated donor is released. In the second step, the sulfuryl group is transferred to the acceptor, regenerating the free enzyme (Figure 3B). The active site consists of the basic amino acids His252, His356, Asn358, Arg374, and Thr501, which probably coordinate the sulfuryl group during the reaction and account for the preferred alkaline pH for catalysis.^36^

The mechanism proposed by Malojčić et al.^36^ is the first model derived from a resolved enzyme structure and may be characteristic of certain homologous ASTs. However, as crystal structures for other ASTs are unavailable, it is not possible to confirm or refute alternative mechanisms, such as that initially suggested by Kim et al.^40^ In both mechanisms, the active site contains a histidine residue that interacts with the donor sulfuryl group and initiates the reaction *via* a ping–pong bi–bi mechanism as described above (Figure 3).

Sequence alignment of putative AST genes from various organisms revealed two distinct classes (clusters) of ASTs.^34^ Cluster I contains ASTs mainly derived from *Proteobacteria* and is characterized by high sequence similarity, while Cluster II includes enzymes originating from *Firmicutes* with lower internal similarity. Another sequence analysis used the two most studied ASTs, *Dh*AST and *Ec*AST, as reference sequences.^35^ This analysis showed that each of these ASTs belongs to a different cluster. Biochemical characterization of active enzymes from both clusters revealed that ASTs from Cluster I (*Ec*AST, *Sb*AST, and *Cf*AST) generally showed higher catalytic efficiency with catechol, while ASTs from Cluster II (*Dh*AST, *Ds*AST, and *Dal*AST) were more effective with phenol as a sulfuryl acceptor. Within each cluster, one enzyme showed superior overall catalytic efficiency: *Cf*AST from Cluster I and *Dal*AST from Cluster II.^61^ Analysis of the sequences of the published ASTs discussed in this review confirmed their division into two distinct clusters (Figure 4). Furthermore, the sequence alignment demonstrated that the catalytic histidine residue identified in *Ec*AST as His436, along with the adjacent residues that assist in binding the sulfuryl moiety, is highly conserved across all known ASTs of both clusters (Figure 5), highlighting their essential role in catalysis. Interestingly, the previously proposed Tyr96 is conserved predominantly in Cluster I ASTs, except for *Bv*AST, which appears to show lower overall similarity to other Cluster I ASTs (Figure 5).

**Figure 4:**
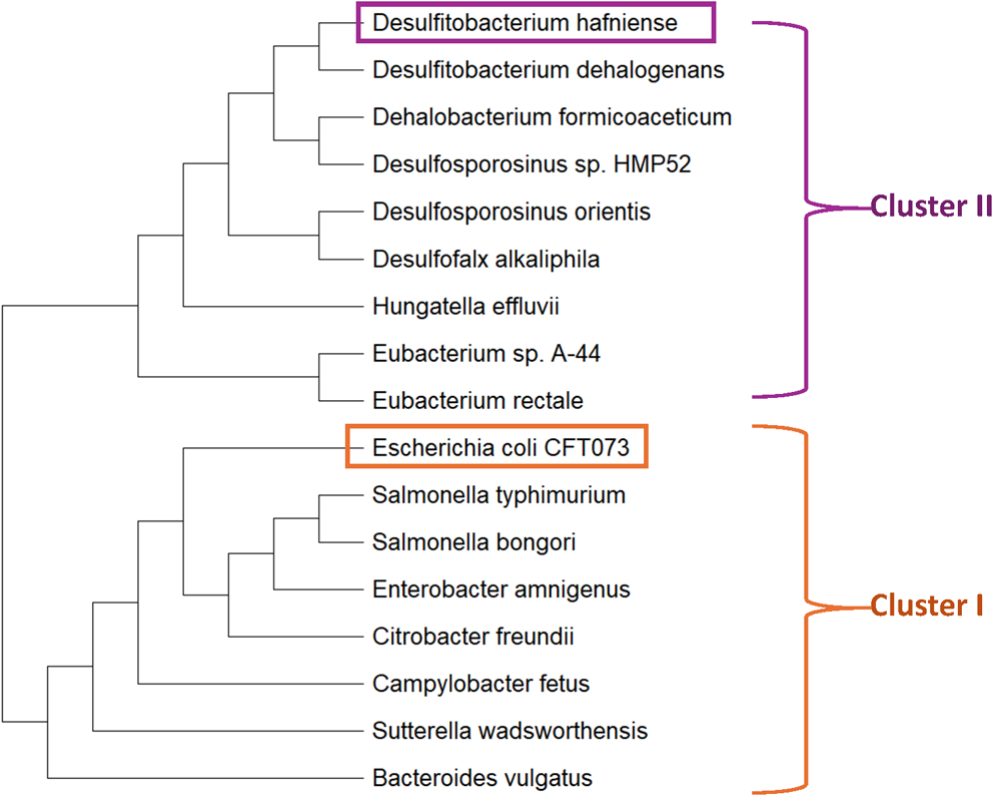
Phylogenetic analysis of published ASTs from representative bacterial species. The analysis identified two distinct AST clusters. Cluster I includes the AST from *E. coli* CFT073 (in an orange frame), the only AST with a resolved structure, whereas Cluster II includes the AST from *D. hafniense* (in a purple frame).

**Figure 5:**
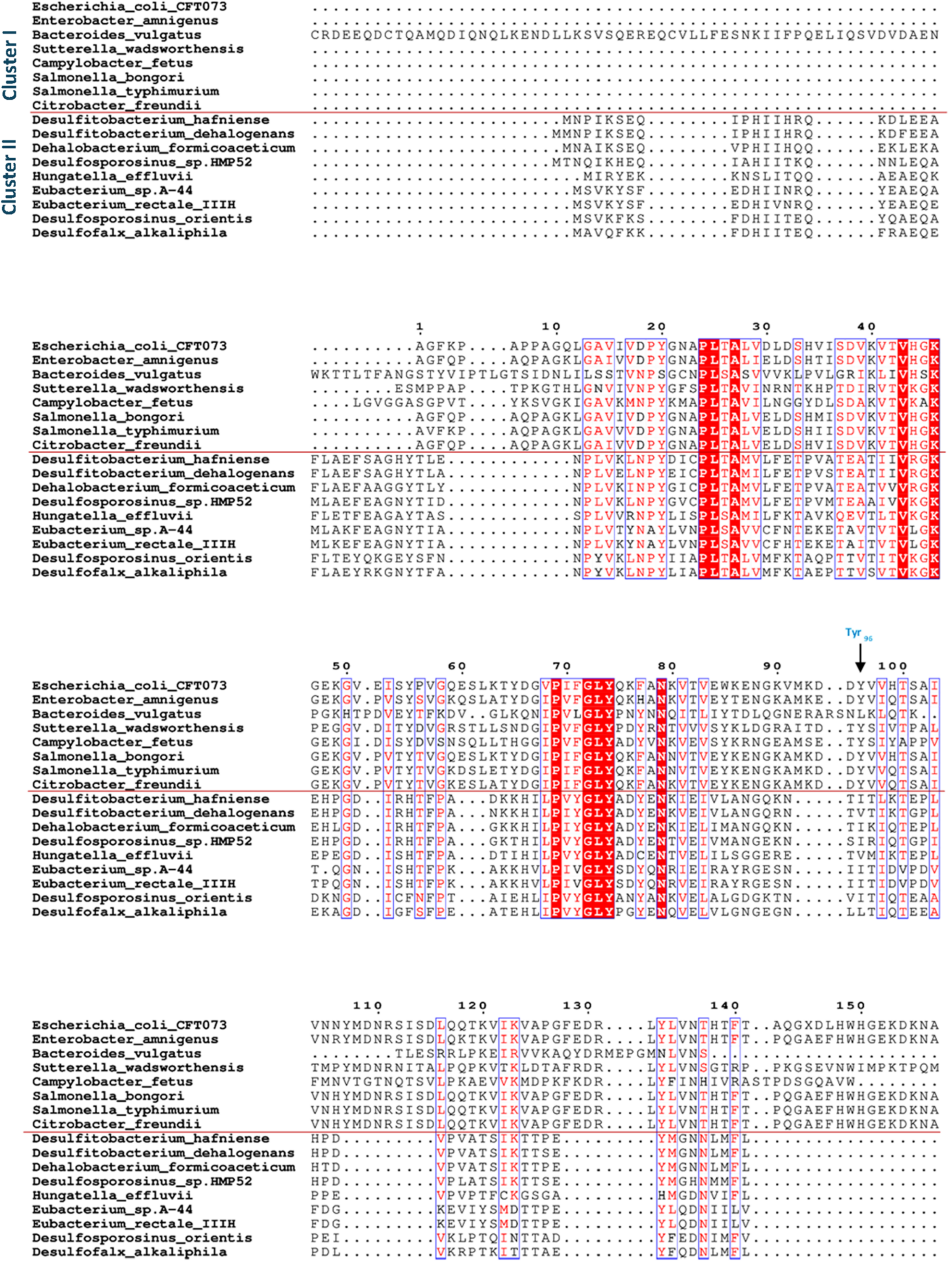

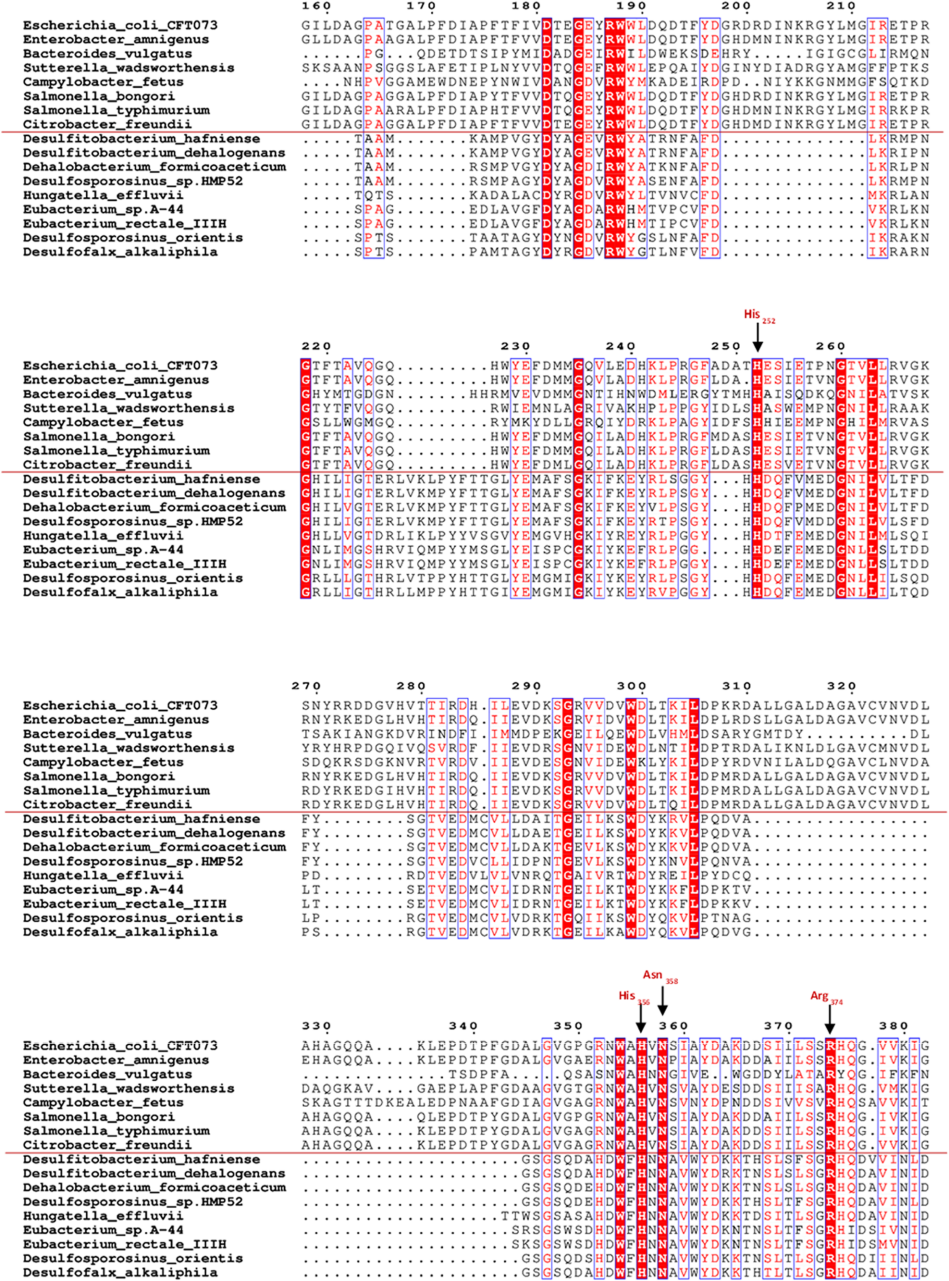

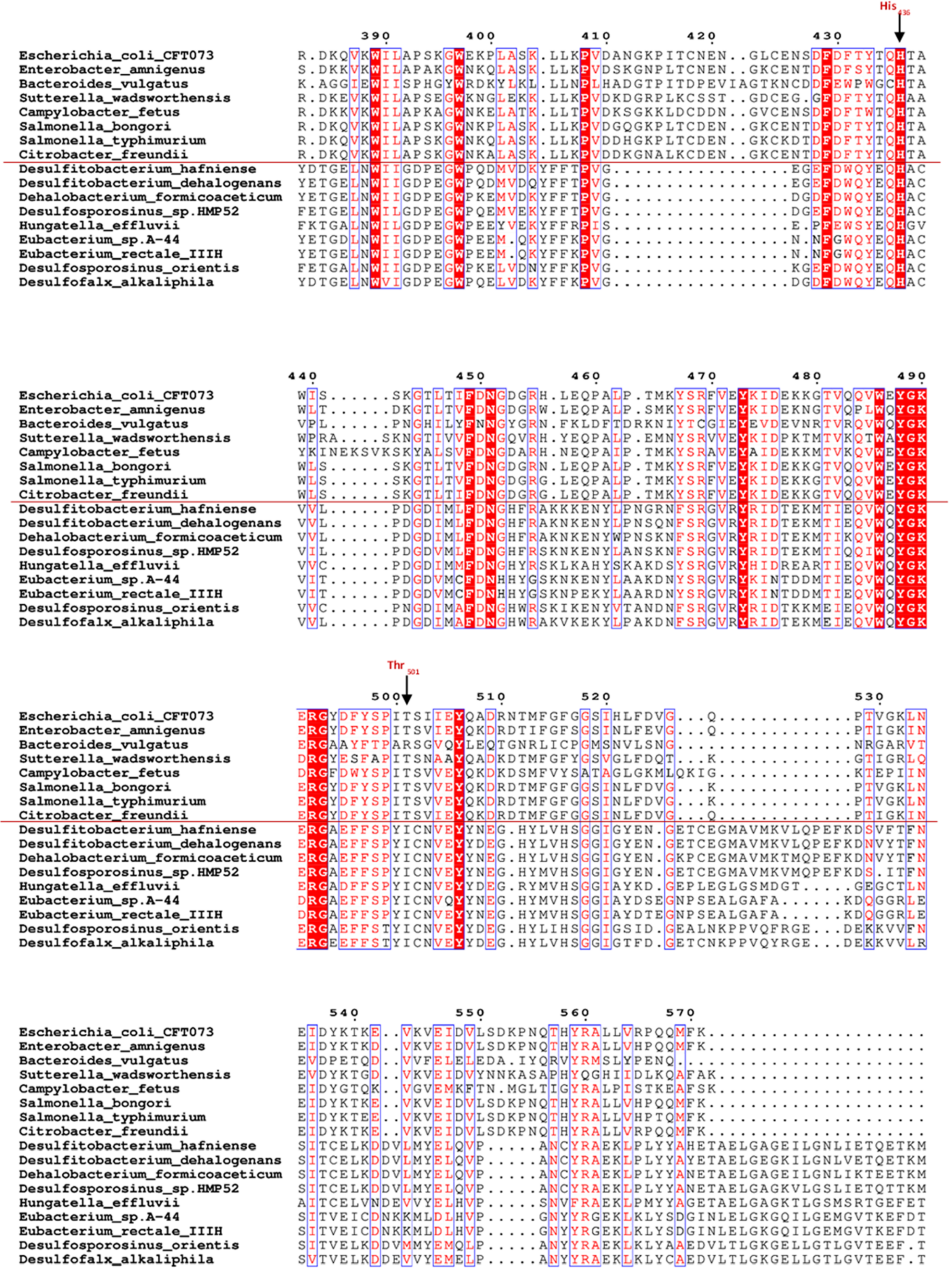
Sequence alignment of known and published ASTs. *Ec*AST was used as the reference sequence, as it is the only AST with a resolved three-dimensional structure. The identified active-site residues of *Ec*AST are conserved across all sequences and highlighted in the figure.

## Substrates of ASTs

ASTs, like other transferases, are specific to both the sulfuryl donor and the acceptor. Therefore, identifying the optimal donor for each acceptor or each acceptor group of individual enzymes is crucial. Most published ASTs largely use the same sets of donors and acceptors. A major shift occurred with the characterization of *Dh*AST and its unique sulfation capabilities.^35,55–57,72^ The potential applications of sulfated alcohols, sugars, and polyphenols have led to extensive testing of the substrate specificity limits of ASTs.

### Sulfuryl Donors of ASTs

Since ASTs were discovered more than 40 years ago, no natural substrate of these enzymes has been identified. Several compounds have been tested as suitable sulfuryl donors for ASTs. A single study on donor substrates showed that only aryl sulfates could serve as donors, while alkyl sulfates were inactive, demonstrating the selectivity of the active site for aromatic groups and excluding PAPS as a potential substrate.^66^ The best-known sulfuryl donors are *p*-nitrophenyl sulfate (*p*NPS) and 4-methylumbelliferyl sulfate (MUS).^33,49,54^ These donors are inexpensive, stable for *in vitro* experiments, and easy to detect. Fluorometric detection of 4-methylumbelliferone released from MUS is approximately 1000 times more sensitive than spectrophotometric detection of *p*-nitrophenol released from *p*NPS at alkaline pH. In addition, the detection limit of fluorimeters is considerably lower than that of visible light spectrophotometers and does not require a specific pH for detection. Therefore, MUS is primarily used for kinetic measurements, while *p*NPS is more commonly used for routine activity determinations and preparative sulfations.^30,40,76^

Among other tested donors (Figure 6), small aromatic compounds such as *p*-nitrocatechol sulfate, *p*-acetylphenyl sulfate, indoxyl sulfate, phenyl sulfate, and α- or β-naphthyl sulfate are primarily found.^35,50,57,66,70^ The position of the sulfuryl group in the donor molecule also affects donor efficiency. For example, β-naphthyl sulfate is almost four times more efficient as a donor for AST from *Haemophilus* K-12 than α-naphthyl sulfate.^45^ Similarly, comparing *o*-, *m*-, and *p*-nitrophenyl sulfates, a clear relationship was observed between the position of the nitro group on the phenol scaffold and enzymatic efficiency.^35^ The measured enzyme efficiency of *p*NPS was 2–8 times higher, and its *K*_M_ value was about 10 times lower than those of the other two nitrophenol isomers.^35^

**Figure 6:**
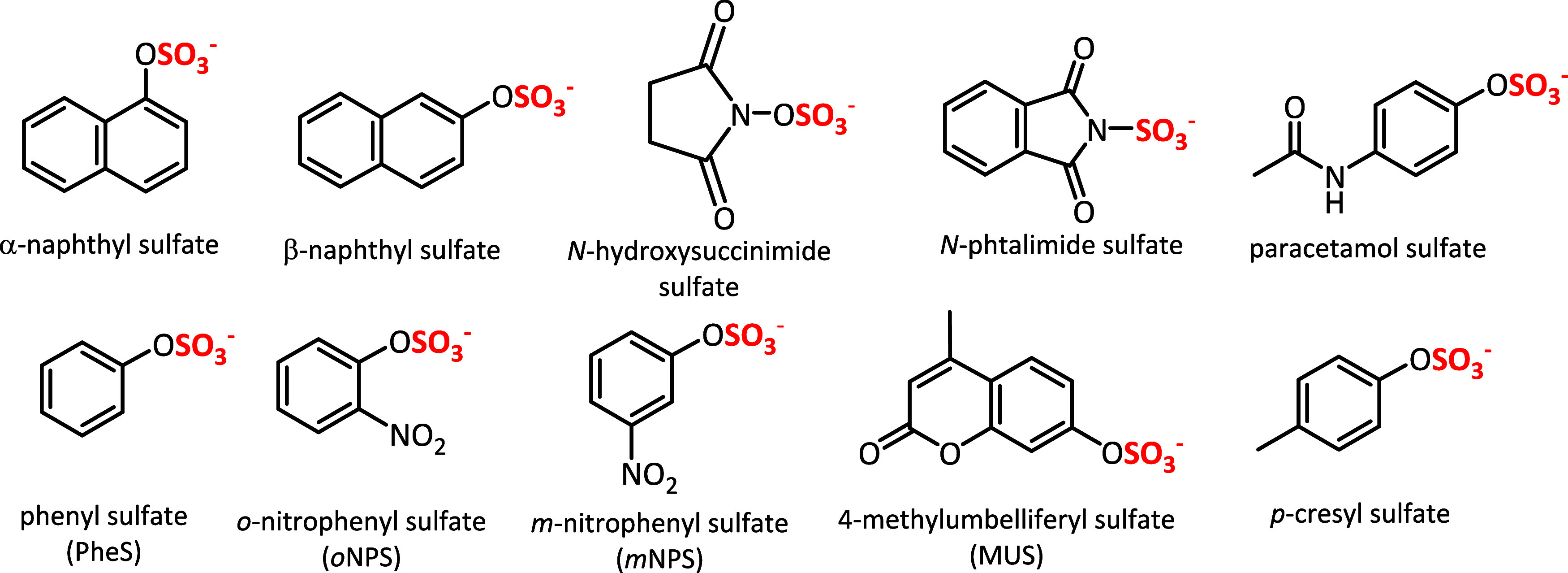
*A*lternative donors for ASTs.

A novel donor, *N*-hydroxysuccinimide sulfate, was introduced by Wever’s group.^57^ Its main advantage, besides its straightforward synthesis, is the harmless and easy-to-remove *N*-hydroxysuccinimide released after hydrolysis. In the sulfations of estradiol and bisphenol A, reactions with the new donor resulted in higher yields than with *p*NPS.^57^ Subsequently, *N*-phthalimide sulfate (Figure 6) was designed to enhance aromatic interactions with AST or the aromatic acceptor. Both donors were evaluated for quercetin sulfation to modulate the sulfate product formation.^8^ Compared with *p*NPS, *N*-hydrosuccinimide sulfate only marginally affected the monosulfate ratio, showed low overall conversion, and produced almost no disulfates.^57^ *N*-Phthalimide sulfate did not yield any detectable quercetin sulfates.^8^

A recent study revealed several noteworthy sulfuryl donors.^66^ Biologically relevant sulfated compounds such as paracetamol sulfate, dopamine sulfate, *p*-coumaryl sulfate, and *p*-cresyl sulfate (Figure 6) were tested as suitable donors for the newly described *Bv*AST with several acceptors. All donors were able to facilitate the sulfation of at least two of the five tested acceptors, and all enabled the sulfation of *p*-nitrophenol.^66^

Although various sulfuryl donors have been tested, *p*NPS remains the preferred choice in sulfation reactions catalyzed by ASTs. It is readily available, chromogenic, easily detectable, and can be removed by extraction with ethyl acetate. Furthermore, the released *p*NP is rarely resulfated by the enzyme.^61^ In contrast to other donors such as sulfated quercetin, phenyl sulfate, and potentially catechol sulfate, which become good acceptors after desulfation (phenol and catechol), *p*NP remains unchanged during the reaction and serves as a reliable indicator of reaction conversion.^30,35^ The standard enzyme activity assay measures the released *p*NP over a defined period in the presence of catechol or phenol as acceptor substrates.^29,34,60–62^ These factors make *p*NPS the optimal donor for activity determination.

All ASTs reported so far have been unable to utilize PAPS as a sulfuryl donor. However, a putative AST encoded by the gene Hoch_5094 from *Haliangium ochraceum* showed unique aryl sulfotransferase activity with this donor.^77^ PAPS is a universal sulfuryl donor in SULT-catalyzed reactions; however, like other cofactors, it requires regeneration. Some PAPS-dependent sulfotransferases, such as aryl sulfotransferase IV from rat liver,^29^ as well as bacterial enzymes from *Haliangium ochraceum* (*Hoc*ST) and *Mycobacterium avium*,^78^ have been shown to regenerate PAPS from PAP within the same catalytic cycle. After desulfation, PAP can act as an acceptor and is resulfated by an external donor distinct from PAPS. Recombinant *Hoc*ST was evaluated for its ability to sulfate alternative acceptors structurally related to PAP. Among a broad range of tested compounds, *Hoc*ST catalyzed the sulfation of phenol, catechol, 4,4′-bisphenol, and four nucleoside triphosphates. These findings suggest potential AST-like activity in *Hoc*ST; however, its acceptor scope and activity levels remain limited compared to PAP sulfation by *p*NPS or the sulfation of other acceptors by PAPS.^77^

### Sulfuryl Acceptors of ASTs

Several compounds have been proposed as natural sulfuryl acceptors of some ASTs. For example, *Ec*AST is suggested to be upregulated in the uropathogenic environment and may act as a virulence factor in urinary tract infections, as only uropathogenic strains were found to carry a gene encoding AST. The periplasmic localization of *Ec*AST and its ability to catalyze sulfuryl transfer between small phenolic compounds suggest that its natural substrates may be present in human urine, where sulfated metabolites are excreted.^79–81^

Caprazamycin, a liponucleoside antibiotic produced by *Streptomyces* sp. MK730–62F2, is synthesized through a pathway involving several enzymes, including Cpz4. Cpz4 is an AST-type sulfotransferase that catalyzes the formation of sulfated caprazamycin derivatives. Although Cpz4 has low sequence homology with other known ASTs, it efficiently transfers sulfuryl groups from donors such as *p*NPS and MUS to phenolic acceptors. Cpz4 plays a well-established physiological role in antibiotic biosynthesis, highlighting its biological significance. Cpz4 exhibits unique substrate specificity, preferentially sulfating the hydroxyl group on the sugar moiety of glycosides rather than phenolic hydroxyl groups.^71^

For most ASTs, natural sulfuryl acceptors have not been identified, and their physiological roles in bacterial cells remain poorly understood. Early studies focused on small phenolic compounds structurally similar to the sulfuryl donors as potential acceptors, and enzyme activity was measured by detecting the released desulfated donor, such as *p*NP or 4-methylumbelliferol. The most frequently tested substrates were phenol, catechol, tyramine, parabens, tyrosine-containing peptides, and other aromatic compounds (Figure 7).^31,41,48,67,68,70^

**Figure 7:**
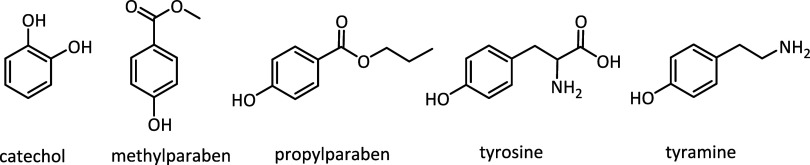
Small phenolic compounds used as sulfuryl acceptors for bacterial ASTs.

A major advance in AST substrate specificity investigations occurred with the sulfation of aliphatic alcohols by *Dh*AST. This recombinant enzyme was able to employ butanol, 2-phenylethanol, glycerol, and other nonphenolic compounds as sulfuryl acceptors, although conversions were lower than with phenolic compounds. The same study also described the sulfation of bulkier and more complex compounds, such as steroids (estrone and estradiol). A preparative reaction with estradiol showed 80% conversion and yielded 79 mg (57%) of sulfated product (17β-estradiol-3-*O*-sulfate) in more than 90% purity (Figure 8 and Table 2).^34^

**Figure 8:**
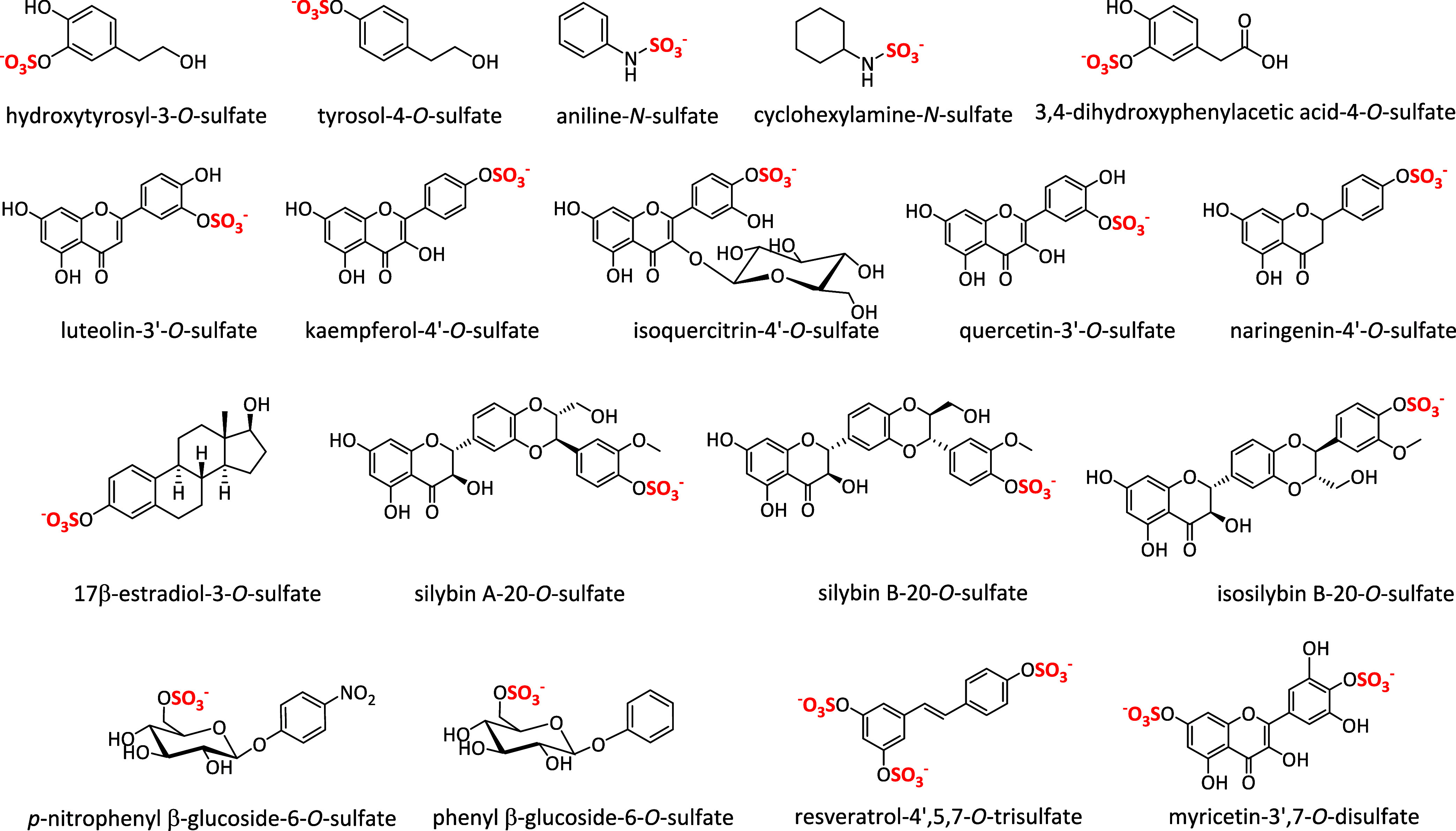
Selected representative products of sulfation by bacterial ASTs.

**Table 2:** Overview of Isolated and Characterized Sulfated Products Synthesized Using Bacterial ASTs^a^

compound	enzyme	isolated yield (mol %)	refs
**Phenols**			
2-Phenylethanol-*O-*sulfate	*Dh*AST	8	^56^
4-Methylcatechol-1/2-sulfates (2:1)		58	^85^
Catechol-1-*O*-sulfate		34	^85^
Hydroxytyrosol-3/4-sulfates (2:1)		49	^73^
Hydroxytyrosol-2′-acetate-3/4-sulfates (1:6)		34	^73^
Phloroglucinol-1-*O*-sulfate		44	^85^
Tyrosol-2′-acetate-4-*O*-sulfate		46	^73^
Tyrosol-4-*O*-sulfate		69	^73^
**Phenolic acids**			
2,3,4-Trihydroxybenzoic acid sulfate	*Dh*AST	8	^85^
3,4-Dihydroxyphenylacetic acid-3/4-*O*-sulfate (6:1)		15	^35,75^
3,4-Dihydroxyphenylpropionic acid-3/4-*O*-sulfate (7:1)		15	^75^
Cinnamic acid-4-*O*-sulfate		85	^72^
Caffeic acid-3/4-*O*-sulfate (69:31)		100	^85^
Gallic acid-3/4-*O*-sulfate (1:2)		19	^35^
Protocatechuic acid-3/4-sulfate (7:3)		4	^85^
**Polyphenols**			
2,2′-Bisphenol-*O*-sulfate	*Dh*AST	46	^72^
2,3-Dehydrosilybin-20-*O*-sulfate		10	^59^
2,3-Dehydrosilybin-7,20-*O*-disulfate		12	^59^
2,3-Dehydrosilychristin-19-*O*-sulfate		0.9	^59^
Ampelopsin-3′-*O*-sulfate		18	^60^
Ampelopsin-4′-*O*-sulfate		22	^60^
Ampelopsin-7,4′-*O*-disulfate		3	^60^
Chrysin-7-*O*-sulfate		1.8	^35^
Hydroxyflavone-6-*O*-sulfate		46	^72^
Luteolin-3′-*O*-sulfate		30	^60^
Luteolin-4′-*O*-sulfate		12	
Luteolin-7,3′-/7,4′-*O*-disulfates (7:1)		12	
Myricetin-3′-*O*-sulfate		42	
Myricetin-4′-*O*-sulfate		3	
Myricetin-7,3′-/3′,4′-*O*-disulfate (1.2:1)		5	
Myricetin-7,4′-*O*-disulfate		4	
Naringenin-4′-*O*-sulfate		51	^13^
Phenylphenol-2-*O*-sulfate		87	^72^
Phloretin-4,4′-*O*-disulfate		19	
Phloretin-4′-*O*-sulfate		21	
Quercetin-3′-*O*-sulfate		15	^8^
Quercetin-3-*O*-sulfate		1	^8^
Quercetin-4′-*O*-sulfate		7	^8^
Quercetin-*O*-disulfate		10	^8^
Resveratrol-3,4′,5-*O*-trisulfate		29	^72^
Resveratrol-3,4′-*O-*disulfate		51	^72^
Resveratrol-3-*O-*sulfate		31	^72^
Resveratrol-4′-*O-*sulfate		7	^72^
Silybin A-20-*O*-sulfate		56	^59^
Silybin B-20-*O*-sulfate		40	
Silychristin-19-*O*-sulfate		32	
Silydianin-19-*O*-sulfate		58	
Taxifolin-3′/4′-*O*-sulfate (80:20)		75	^30^
Kaempferol-3,4′-*O*-disulfate	*Dal*AST	-	^61^
Kaempferol-4′-*O*-sulfate		21	
Kaempferol-7,4′-*O*-disulfate		13	
Kaempferol-7-*O*-sulfate		-	
**Steroids**			
17β-Estradiol-3-*O*-sulfate	*Dh*AST	57	^34^
**Glycosides/carbohydrates**			
Isoquercitrin-4′-O-sulfate	*Dh*AST	69	^30^
Phenyl β-d-glucopyranoside-6-*O*-sulfate		2.3	^56^
*p*-Nitrophenyl β-d-glucopyranoside-6-*O*-sulfate		30	
*p*-Nitrophenyl 2-acetamido-2-deoxy-β-d-glucopyranosid*e*-6-*O*-sulfate		2	
*p*-Nitrophenyl glycerol-3-*O*-sulfate (*R*)		4	
Rutin-4′-*O*-sulfate		53	^30^

a DhAST—aryl sulfotransferase from *Desulfitobacterium hafniense*, *Dal*AST—aryl sulfotransferase from *Desulfofalx alkaliphile*.

The sulfation of aliphatic acceptors by *Dh*AST opened new pathways for sulfating other nonphenolic molecules, including sugars and other carbohydrates. Sulfated carbohydrates are important building blocks and serve as substrates for many glycosyltransferases,^82^ whose reactions produce more complex oligosaccharides. Sulfation of glucose and glycerol was previously observed with *Dh*AST, but with low conversion rates.^34^ Attaching aromatic groups, such as phenol or *p*NP, to these substrates enhanced their sulfation. This substrate modification enabled the synthesis of several sulfated carbohydrates with yields up to 30% (Figure 8 and Table 2).^56^ Another way to improve the enzyme catalytic performance is through protein engineering. Directed enzyme evolution was applied to *Dh*AST to increase its activity toward saccharide compounds. The resulting mutant variant, ASTB-V1, carrying the amino acid substitution Val579Asp, demonstrated higher activity with mono-, di-, and trisaccharides compared to the wild-type enzyme, with up to a 5.4-fold activity increase in the sulfation of glucose.^83^

Aryl sulfotransferase from *D. hafniense* is not the only AST that was able to catalyze the sulfation of sugars. A recent study of putative aryl sulfotransferases revealed the AST from *Hungatella effluvii* (*Hef*AST in this review; 58.5% sequence homology to *Dh*AST) that efficiently sulfated *N*-acetylglucosamine (GlcNAc). *Hef*AST exhibited an approximately six times higher activity for GlcNAc than *Dh*AST, which made it a promising tool for the synthesis of sulfated sugars and oligosaccharides.^62^

Another important group of AST acceptors comprises polyphenols, which are structurally bulkier and highly diverse. This structural diversity enables an almost unlimited range of potential AST substrates. The flavonolignans silybin, isosilybin, silychristin, silydianin, and others are natural bioactive compounds found in milk thistle (*Silybum marianum*) seeds, known for their potent antioxidant, hepatoprotective, and other health benefits.^84^ These compounds naturally occur as a mixture of diastereoisomers. *Dh*AST enabled a highly effective regioselective sulfation of these flavonolignans, producing optically pure sulfated metabolites of both silybins and isosilybins: silybin A-20-*O*-sulfate, silybin B-20-*O*-sulfate, isosilybin A-20-*O*-sulfate, and isosilybin B-20-sulfate, using *p*NPS as the sulfuryl donor (Table 1). Sulfation of such complex compounds suggests that the active site is not spatially limited for the acceptor substrate.^55^ Interestingly, silybin A and B were also tested as substrates for a mammalian PAPS-dependent aryl sulfotransferase IV (AstIV) from rat liver.^29^ This enzyme was able to sulfate only silybin B at the position C-20, while silybin A remained unreacted (Figure 8). The sulfation of silybin B with AstIV was slower than with *Dh*AST, and the product yield was more than 50% lower than that of *Dh*AST-mediated sulfation. In a later study, using *Dh*AST crude cell extract resulted in higher yields in the sulfation of flavonolignans. In this study, 2,3-dehydrosilybin-20-*O*-sulfate, 2,3-dehydrosilybin-7,20-*O*-disulfate, silychristin-19-*O*-sulfate, 2,3-dehydrosilychristin-19-*O*-sulfate, and silydianin-19-*O*-sulfate were prepared, along with pure silybin A-20-*O*-sulfate and silybin B-20-sulfate.^59^

Polyphenolic substrates differ mainly in the number and position of hydroxyl (−OH) groups in their scaffold. These structural differences determine their properties and functions; therefore, the specificity and catalytic efficiency of ASTs can vary between substrates. Enzyme regioselectivity also affects the order of subsequent sulfation steps, resulting in the formation of di- and trisulfated products.^60,61,72^ In these cases, the regioselectivity of the reaction is especially important. In contrast to flavonolignans, which have the B ring attached to the D ring and are sulfated on E ring, in flavonoids, the preferred sulfation sites are the hydroxyl groups at the C-3′ and C-4′ positions. This produces isomeric products that cannot always be separated during initial analytical detection.^85^ Once all primary preferred sulfation sites are occupied, the next common site of sulfation is C-7,^35^ leading to the formation of disulfates. Sulfation at adjacent positions is generally not observed, probably due to steric limitations.^62^ Trisulfates have been reported as products of resveratrol sulfation.^72^ Myricetin has three hydroxyl groups on the B ring that are close to each other, which leads to the formation of two main disulfate isomers: myricetin-3′,7-*O*-disulfate and myricetin-4’,7-*O*-disulfate. The first isomer can subsequently be sulfated at the C-5′ position to form a trisulfate, but the concentration of trisulfates in reaction mixtures was low.^60^

The sulfation capacity of *Dh*AST was compared to the PAPS-dependent aryl sulfotransferase AstIV for their ability to sulfate quercetin, its glycosylated derivatives (isoquercitrin and rutin), and taxifolin. The eukaryotic AstIV was able to sulfate only the flavonoids quercetin and taxifolin and showed no activity with the glycosides. In contrast, *Dh*AST selectively sulfated isoquercitrin and rutin at the C-4′ position. Taxifolin was mainly sulfated at the C-4′ position, although a small amount of the C-3′ isomer was also formed. For quercetin, sulfation occurred mainly at the C-3′ position, but a smaller amount of the C-4′ isomer was also produced. In a later study, quercetin-3-*O*-sulfate, quercetin-7,3′-*O*-disulfate, quercetin-7,4′-*O*-disulfate, and quercetin-3′,4′-*O*-disulfate were also described as minor products.^8^ In addition, sulfated quercetin was shown to act as a sulfuryl donor in the sulfation of catechol. Among the isomers, the 3′-*O*-sulfated quercetin exhibited slightly higher donor efficiency compared to the 4′-*O*-sulfated form.^30^

A recent study on previously reported and new ASTs from both proposed clusters identified luteolin and kaempferol (Figure 8) as the preferred acceptors of the tested ASTs.^61^ Reactions with these two compounds showed the highest conversion rates for all tested ASTs from both clusters. The same study identified one AST from each cluster with the highest sulfation efficiency for the tested (poly)­phenolic acceptors. ASTs from *Desulfofalx alkaliphile* (*Dal*AST; Cluster II related to *Dh*AST) and *Campylobacter fetus* (*Cf*AST; Cluster I related to *Ec*AST) demonstrated greater capability for sulfating the tested polyphenols than the known and widely used *Ec*AST and *Dh*AST. This work also resulted in the first preparation of sulfated derivatives of kaempferol (Table 2).^61^

Generally, sulfotransferases transfer a sulfuryl group not only to free hydroxyls but also to amines. These reactions have been observed with SULTs but have not been described with ASTs so far. Recent screening of *Dh*AST and several newly identified ASTs with amine-containing compounds showed that these enzymes exhibited activity with at least one of the substrates, aniline or cyclohexylamine (Figure 8), with a preference for the aromatic substrate. This finding indicates that ASTs can sulfate amino groups even when the acceptor molecule lacks hydroxyl groups.^62^

Some flavonoids are difficult to sulfate; for example, chrysin is often considered an inert substrate because it lacks hydroxyl (−OH) groups on ring B, which is the preferred position for the sulfation in flavonoids. Despite this, when chrysin was treated with the enzyme *Dh*AST, a sulfated product was obtained, albeit in very low yield (1.8%).^35^ A more recent screen of new aryl sulfotransferases (ASTs) found that *Dal*AST and *Cf*AST can convert chrysin at rates of up to 9%,^61^ but the sulfated product is unstable, which limits its preparative use.^35^

Other challenging substrates are phenolic acids. Compared to flavonoids and other polyphenolic compounds, phenolic acids are present in plants in relatively small amounts. Flavonoid absorption into the human bloodstream occurs primarily in the gut, where the intestinal microbiota metabolize these compounds to low-molecular-weight phenols and phenolic acids.^86,87^ As a result, phenolic acids become predominant metabolites in the bloodstream and undergo further biotransformation.^88^ Some phenolic acids pose a challenge for enzymatic sulfation mainly because their carboxyl groups can lower the reaction pH. As ASTs are primarily active in an alkaline environment (pH 8–9), these substrates can hinder sulfation. Indeed, screening with various sulfuryl donors and challenging acceptors such as chrysin and gallic acid confirmed the crucial role of both the number and position of hydroxyl groups for successful sulfation. Phenolic acids with two or more hydroxy groups were sulfated by some ASTs,^35,61,66,75^ while monohydroxy phenylacetic and phenylpropionic remained unreacted.^75^ Analysis of the reaction mixtures with these acids showed a limited release of *p*NP at the beginning, after which the reaction state remained unchanged over time. Moreover, the acids can form complexes with buffer salts, further complicating catalysis and analysis of the reaction mixture.^75^ However, a monohydroxy *p*-coumaric acid was reported as a suitable acceptor substrate for *Dh*AST and *Bv*AST.^66,72^ This acid contains a double bond conjugated to the aromatic ring, which probably affects the ASTs’ affinity for it.

In conclusion, this review highlights the remarkable versatility of bacterial aryl sulfotransferases in synthetic applications and sets the stage for future advances in enzymatic sulfation, particularly for phenolic compounds, including flavonoid derivatives and other biologically relevant molecules. ASTs represent a promising yet underexplored group of enzymes. They are robust, cofactor-independent, and exhibit broad substrate specificity, making them highly attractive tools for *in vitro* biocatalysis. Advances in recombinant expression systems have enabled the efficient production of these enzymes in *E. coli*, simplifying their purification and characterization. The physiological role of ASTs in bacteria remains poorly understood, as their natural substrates and potential inhibitors have not yet been identified. The acceptor specificity of *Dh*AST and recently identified ASTs has expanded considerably over the past decade. These enzymes are more efficient than previously known ones, and their catalytic potential appears virtually limitless. The discovery of novel ASTs, combined with the exploration of new substrates, will contribute to the development of a detailed and comprehensive library of sulfated metabolites and their synthesis *in vitro*. Such products can serve as valuable analytical standards and tools for biological testing to better understand their effects on the human body.

Future studies expanding the library of characterized ASTs from both Cluster I and Cluster II are likely to reveal new catalytic features and broaden their potential applications. Following the demonstrated sulfation of polyphenols, research may now focus on larger and more complex acceptors, such as antibiotics. In addition, *in vitro* production of sulfated sugars and oligosaccharides, including glycosaminoglycans (GAGs), may represent an important step toward understanding their biological properties and functions in various disorders.^89^ Importantly, resolving the crystal structures of the effective ASTs, such as *Dh*AST and *Dal*AST, would open the path to rational enzyme engineering and substrate tailoring for specific research or synthetic purposes, which should be the major focus of the current investigations of ASTs.
